# Beyond Neuronal Microtubule Stabilization: MAP6 and CRMPS, Two Converging Stories

**DOI:** 10.3389/fnmol.2021.665693

**Published:** 2021-05-05

**Authors:** Camille Cuveillier, Benoit Boulan, Charlotte Ravanello, Eric Denarier, Jean-Christophe Deloulme, Sylvie Gory-Fauré, Christian Delphin, Christophe Bosc, Isabelle Arnal, Annie Andrieux

**Affiliations:** Univ. Grenoble Alpes, Inserm U1216, CEA-IRIG, CNRS, Grenoble Institut Neurosciences, Grenoble, France

**Keywords:** microtubule, microtubule-associated-proteins, actin, neuron, synapse, psychiatric disease, cognition

## Abstract

The development and function of the central nervous system rely on the microtubule (MT) and actin cytoskeletons and their respective effectors. Although the structural role of the cytoskeleton has long been acknowledged in neuronal morphology and activity, it was recently recognized to play the role of a signaling platform. Following this recognition, research into Microtubule Associated Proteins (MAPs) diversified. Indeed, historically, structural MAPs—including MAP1B, MAP2, Tau, and MAP6 (also known as STOP);—were identified and described as MT-binding and -stabilizing proteins. Extensive data obtained over the last 20 years indicated that these structural MAPs could also contribute to a variety of other molecular roles. Among multi-role MAPs, MAP6 provides a striking example illustrating the diverse molecular and cellular properties of MAPs and showing how their functional versatility contributes to the central nervous system. In this review, in addition to MAP6’s effect on microtubules, we describe its impact on the actin cytoskeleton, on neuroreceptor homeostasis, and its involvement in signaling pathways governing neuron development and maturation. We also discuss its roles in synaptic plasticity, brain connectivity, and cognitive abilities, as well as the potential relationships between the integrated brain functions of MAP6 and its molecular activities. In parallel, the Collapsin Response Mediator Proteins (CRMPs) are presented as examples of how other proteins, not initially identified as MAPs, fall into the broader MAP family. These proteins bind MTs as well as exhibiting molecular and cellular properties very similar to MAP6. Finally, we briefly summarize the multiple similarities between other classical structural MAPs and MAP6 or CRMPs.In summary, this review revisits the molecular properties and the cellular and neuronal roles of the classical MAPs, broadening our definition of what constitutes a MAP.

## Introduction

Microtubule-Associated Proteins (MAPs) were discovered in the context of the study of microtubule (MT) stability in neurons during the 1970s (Weingarten et al., 1975; Sloboda et al., 1976). Indeed, in neurons, MTs composed of α- and β-tubulin heterodimers forming 25 nm wide hollow tubes can either exhibit dynamic properties with phases of polymerization/depolymerization (Mitchison and Kirschner, 1984), or be particularly stable with a slow tubulin turnover (Okabe and Hirokawa, 1990; Li and Black, 1996). In testimony to their stability, neuronal MTs can resist conditions usually causing MT depolymerization, such as exposure to tubulin poison (nocodazole) or to cold temperatures (Webb and Wilson, 1980; Brady et al., 1984; Baas and Black, 1990). The search for the neuronal effectors leading to this stability has long been a focus of research. Various factors were shown to modulate neuronal MT stability, including: (1) the nature of tubulin isotypes, (2) post-translational tubulin modifications, and (3) binding of MAPs (Baas et al., 2016).

The nature of tubulin isotypes was clearly demonstrated in mice, where α and β tubulin are each coded by eight genes (Findeisen et al., 2014; Hausrat et al., 2020). The different isoforms combine to produce MTs with distinct dynamic parameters. For example, the βIII/βII tubulin MT isoform displays antagonist effects on dynamicity/stability (Panda et al., 1994; Pamula et al., 2016; Vemu et al., 2016; Ti et al., 2018).

Post-translational modifications of tubulin—including tyrosination, polyamination, polyglutamylation, and acetylation—were shown to modulate MT stability by altering their dynamic properties (Moutin et al., 2020), resistance to cold exposure (Song et al., 2013), sensitivity to severing enzymes (Lacroix et al., 2010; Valenstein and Roll-Mecak, 2016), and flexibility (Portran et al., 2017; Xu et al., 2017). Finally, the so-called structural MAPs which bind throughout the MT lattice also influence MT stabilization. As indicated above, these structural MAPs were first discovered in the 1970s, when they were found to be associated with purified brain tubulin preparations. The group includes MAP1, MAP2, MAP4, Tau, and MAP6 (also known as STOP; Cleveland et al., 1977; Herzog and Weber, 1978; Huber and Matus, 1984; Margolis et al., 1986; Job et al., 1987; Chapin and Bulinski, 1994). Other members were identified more recently: DCX, MAP7, and MAP9 (Gleeson et al., 1999; Yadav et al., 2014; Monroy et al., 2020).

Since the initial discovery of these MAPs based on their ability to bind and stabilize MTs, studies have increasingly pointed toward a wide array of other cellular functions (Dehmelt and Halpain, 2005; Morris et al., 2011; Dent and Baas, 2014; Bodakuntla et al., 2019). Thus, structural MAPs have been shown to: regulate actin cytoskeleton dynamics; be amenable to post-translational modifications which target them to membrane compartments; interact with a huge number of partners which are then involved in neuroreceptor homeostasis and signaling cascades. These additional abilities stem from molecular features including actin-binding domains, stretches of cysteine residues for palmitoylation-driven membrane association, and Proline-Rich-Domain (PRDs) to mediate binding to SH3-containing signaling proteins.

In this review, we will present results obtained over almost 40 years of research on MAP6 proteins to illustrate the wide range of MT-dependent and -independent molecular properties that MAPs can exhibit. Using the Collapsin Response Mediator Proteins (CRMPs) as an example, we will show how other proteins not initially identified as MAPs also fulfill molecular and cellular functions initially attributed to the classical structural MAPs. We will also discuss the multiple cellular and physiological roles of MAPs in neurons and in the central nervous system. The review will, in particular, illustrate the crucial implication of MAPs in neuronal plasticity and cognition in accordance with their dysfunctions in neuropsychiatric diseases. Overall, we aim at demonstrating that the initial definition of classical MAPs (i.e., MT binding and stabilization) should now encompass the MT-dependent and -independent functions of these proteins.

## Map6 Protein Is A Multi-Functional Protein

**Figure 1:**
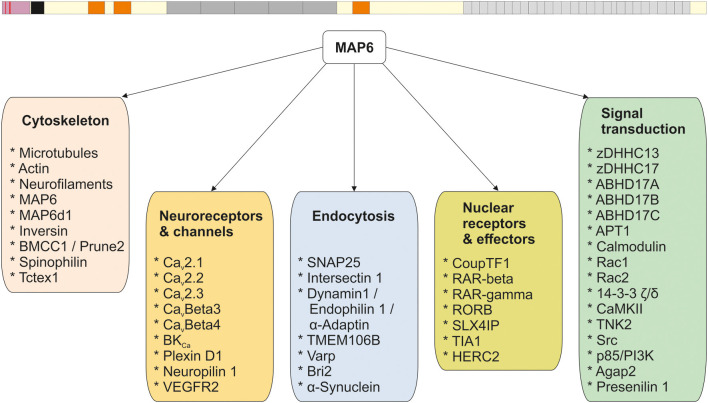
MAP6 protein and its interactors. All the known MAP6 interactors (see Supplementary Table 1) are presented and grouped based on their functions.

**Figure 2:**
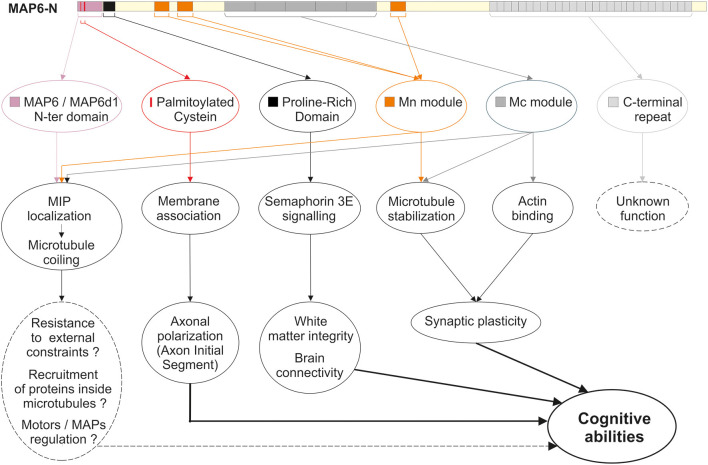
MAP6 domains and functions. Schematic representation of MAP6, color-coded arrows indicate the different domains identified so far: the N-terminal domain shared with MAP6d1 protein (violet box); the three palmitoylation sites (red bars); the proline-rich domain involved in transduction of Semaphorin 3A signals (black box); the three microtubule-stabilizing Mn modules (orange boxes); the tandem repeats corresponding to microtubule-stabilizing Mc modules (dark gray boxes); the C-terminal repeats (light gray boxes). Below the representation, the corresponding roles of these domains are indicated, covering molecular and cellular roles, and tissues and integrated functions.

Like the other structural MAPs, MAP6 protein was initially identified thanks to its ability to interact with tubulin/MTs. Subsequent studies identified a large number of MAP6 partners related to various cellular functions including neuroreceptor homeostasis, endocytosis, nuclear function, and signaling pathways. The different partners are summarized in Figure 1, and further details are provided in Supplementary Table 1. In the following sections, the contribution of each MAP6 sub-domain to its molecular, cellular, and physiological functions are detailed. A summary of MAP6 domains and their roles is presented in Figure 2.

### MAP6 Binds and Stabilizes Microtubules

#### Discovery of MAP6 as a Microtubule (MT)-Associated Protein

The exceptional stability of brain-extracted MTs when exposed to cold was first described almost half a century ago (Brinkley and Cartwright, 1975; Lee et al., 1975; Webb and Wilson, 1980). Subsequent work led to the identification of several polypeptides that co-purify with cold-stable MTs (Job et al., 1981, 1982; Margolis and Rauch, 1981; Pabion et al., 1984) and are retained on calmodulin-affinity columns (Job et al., 1982; Pirollet et al., 1983). The unique ability of these polypeptides to confer cold stability on MTs led Job and collaborators to call them STOP proteins, for Stable-Tubule-Only Polypeptides (they were later renamed MAP6 proteins). The MT stabilization properties of MAP6 are Ca^2+^-calmodulin sensitive (Job et al., 1981; Pirollet et al., 1983, 1992a,b) and the 145-kDa STOP isoform from the rat brain was shown to confer a super-stable state on MTs, in a dose-dependent manner (Job et al., 1987).

#### Molecular Cloning of MAP6 and Identification of MAP6 Isoforms

Bosc et al. (1996) first cloned MAP6 cDNA by immuno-screening of a DNA expression library with MAP6 monoclonal antibodies (Pirollet et al., 1989). These experiments provided clear identification of MAP6 and made further studies to gather specific information on the protein possible. MAP6 isoforms are restricted to vertebrates, where they are expressed in several tissues (Pirollet et al., 1989; Bosc et al., 2001). In mice, a single four-exon gene, Map6 (formerly Mtap6), produces several isoforms of MAP6 proteins as a result of RNA splicing and the use of alternative promoters (Bosc et al., 1996; Denarier et al., 1998a; Aguezzoul et al., 2003). Murine neurons express MAP6-E (MAP6-Early, formerly E-STOP, apparent MW 79 kDa) from early developmental stages to adulthood at constant expression levels, whereas expression of the longest isoform MAP6-N (MAP6-Neuronal, formerly N-STOP, apparent MW 120 kDa) increases between birth and adulthood (up to 10-fold higher expression than MAP6-E in adults) (Guillaud et al., 1998; Galiano et al., 2004; Tortosa et al., 2017). These two variants can be found associated with MTs in neurons and stabilize neuronal MTs when exposed to cold or nocodazole (Pirollet et al., 1989; Margolis et al., 1990; Bosc et al., 1996; Guillaud et al., 1998; Andrieux et al., 2002). Glial cells also express some MAP6 isoforms, with MAP6-O (apparent MW 89 kDa) found in oligodendrocytes, where they provide resistance to both cold and nocodazole. In contrast, astrocytes express MAP6-A (apparent MW 66 kDa), which only provides cold resistance (Galiano et al., 2004). Like MAP6-A, the ubiquitous MAP6-F isoform (apparent MW 42 kDa) provides only cold resistance (Denarier et al., 1998b).

#### Identification of MAP6 MT-Binding Domains and Their Role in MT Stabilization

The identification of 12 calmodulin-binding domains on MAP6-N revealed the existence of three domains that partially overlap some of the calmodulin-binding motifs. These three domains are known as Mn modules (Mn1–3, Figure 2) and stabilize MTs against both cold and nocodazole-induced depolymerization (Bosc et al., 2001). Mn1 and Mn2 modules were shown to reproduce the function of full-length MAP6-N with regard to MT stabilization, both *in vitro* and *in cellulo* (Bosc et al., 2001; Lefevre et al., 2013). Thus, these modules may stabilize MTs exposed to cold and nocodazole by forming bridges with adjacent tubulin heterodimers either between protofilaments, or longitudinally within the same protofilament (Lefevre et al., 2013).

In addition to its Mn modules, MAP6-N contains central repeats, or Mc modules, each of which encompass a calmodulin-binding domain (Figure 2). As indicated above, MAP6 proteins are found only in vertebrates, but Mc modules are further restricted. Thus, these domains have only been identified in mammals and are absent from MAP6 homologs expressed in fish, frogs, lizards or birds (Bosc et al., 2001, 2003). Among mammals, the number of Mc modules varies depending on the species and/or individual (4–6 in non-inbred rats, 3–6 in mice depending on the strain, and only 1 in higher primates) (Bosc et al., 2003). Mc modules have been shown to be responsible for conferring cold-stability of MTs in cells, even though they display no MT-binding capacity at physiological temperatures (Denarier et al., 1998b; Delphin et al., 2012). *In vitro* studies demonstrated that cold temperatures induce conformational changes in the Mc modules which allow them to interact with MTs (Delphin et al., 2012). Thus, Mc modules behave like cold sensors, stabilizing MTs when temperatures drop, for example during hibernation or torpor.

The high abundance of calmodulin-binding domains overlapping the Mn and Mc modules in MAP6 hints that MAP6 binding to MTs is likely to be tightly regulated in cells. Indeed, it was shown *in vitro* that Ca^2+^-Calmodulin (CaM) binding to MAP6 occurs in an unusual manner (Bouvier et al., 2003) and prevents MAP6 binding to MTs (Lefevre et al., 2013). MAP6/MTs interaction is also prevented by phosphorylation of MAP6 by CAMKII and favors MAP6/F-actin interaction in neurons (Baratier et al., 2006). Based on the available experimental data and as proposed by Ramkumar and collaborators (Ramkumar et al., 2018), the regulation of MAP6 by Ca^2+^-CaM in dendritic spines might follow this sequence: upon synaptic activity, the Ca^2+^-CaM complex forms, detaches MAP6 from adjacent MTs and activates CAMKII (Fink and Meyer, 2002). When Ca^2+^ level decreases, CaM is released from MAP6 allowing its phosphorylation by CAMKII. Then phosphorylated MAP6 is unable to re-associate with MTs but rather binds and stabilizes the synaptic F-actin (Baratier et al., 2006; Peris et al., 2018).

Recently, in cell-free systems, recombinant MAP6-N was shown to exert some stabilizing effects on MT dynamics by promoting rescue (Cuveillier et al., 2020), although the precise contribution of the various MAP6-N MT-binding domains (Mn and/or Mc modules) remains to be clarified. Indeed, the specific role of MAP6-related MT stabilization in developing neurons remains unclear. For example, MT-dependent parameters of neuronal differentiation and morphology (e.g., neurite elongation and branching, axonal polarization) are not dramatically altered in neurons expressing reduced MAP6 levels. Indeed, MAP6 deficient neurons exhibited only moderate morphological defects with a slight increase of the axonal length and a decreased spine density (Andrieux et al., 2002, 2006; Peris et al., 2018; Boulan et al., 2020), as well as a reduced growth cone size and an increased dendrite branching (Schwenk et al., 2014; Qiang et al., 2018). Those defects are not striking possibly due to compensatory mechanisms by other MAPs. Indeed, double-KO neurons for Tau/MAP1B or MAP2/MAP1B display stronger alterations of neuronal differentiation (Takei et al., 2000; Teng et al., 2001) than single KO neurons (Harada et al., 1994; Takei et al., 1997; Gonzalez-Billault et al., 2001). In this respect, an increased expression of Tau has been observed when MAP6 expression is knocked-down even though Tau and MAP6 role in MT stabilization are not similar in neurons (Qiang et al., 2018). In developing neurons, MAP6 was found enriched in the proximal part of the future axon (Tortosa et al., 2017) and a recent proteomic analysis pointed out MAP6 as a specific component of the AIS (Hamdan et al., 2020). MAP6-N localization in the proximal part of axons has been shown to be protective toward the formation of axonal varicosities induced by mechanical stress (Gu et al., 2017). Still, the exact relationships between MAP6-dependent MT stabilization, axonal polarization and AIS functions remains elusive.

#### MAP6 Belongs to a Family of Proteins

Although most structural MAPs exhibit repeated MT-binding motifs, the repeated motifs in MAP6—its Mn and Mc modules—are unique, and share no homology with MT-binding domains found in Tau or MAP1B (Bosc et al., 1996). However, bioinformatic analysis of the MAP6 sequence has revealed three proteins that share homology in MAP6 functional domains.

The first one is MAP6d1, for MAP6-domain-containing protein 1 (formerly SL21 for 21-kDa STOP-like protein). MAP6d1 is expressed at high levels in the brain and shares two major similarities with MAP6: 80% sequence homology in its N-terminal domain, and 72% sequence homology with the Mn3 module of MAP6 (Bosc et al., 2001; Gory-Fauré et al., 2006). Since the N-terminal domain of both proteins contains the main calmodulin-binding site (Bosc et al., 2001), its functional role might be important.

The original MAP6 family was enlarged following the discovery of homologs of SAXO proteins (SAXO1 and SAXO2 in mammals, formerly named FAM154A and FAM154B, respectively). These proteins were originally identified in the protozoan Trypanosoma brucei and in ciliated/flagellated cells (Dacheux et al., 2012). Like neuronal MTs, cilia and flagella are characterized by a high level of MT stability displaying resistance to cold- and nocodazole-induced depolymerization. The human isoform hSAXO1 is ubiquitously expressed and specifically associates with centrioles, basal bodies and cilia (Dacheux et al., 2015). Little is known about SAXO2 beyond the fact that its expression appears to be enriched in ciliated cells.

SAXO proteins share homologies with the Mn modules of MAP6/MAP6d1, and their N-terminal sequence, although distinct, is also rich in cysteines (Dacheux et al., 2012). Interestingly, 80% of hSAXO1 consists of 12 tandem Mn modules that, like the equivalent modules in MAP6 and MAP6d1, are involved in MT binding and cold stabilization. The high number of Mn modules is important since hSAXO1 overexpression results in an increased primary cilia length in RPE1 cells, through a mechanism requiring the Mn modules (Dacheux et al., 2015).

#### MAP6 Is a Microtubule Inner Protein (MIP)

Cryo-EM experiments revealed neuronal MTs to contain visible intraluminal densities (Burton, 1984; Garvalov et al., 2006; Atherton et al., 2018). These densities correspond to Microtubule Inner Proteins (MIPs), the molecular identity of which was totally unknown until MAP6 was identified as a MIP (Cuveillier et al., 2020). The capacity of MAP6 to enter the lumen of MTs was demonstrated using cell-free systems in which MTs were copolymerized with MAP6, yielding MTs with regularly spaced intraluminal densities. When using MTs derived from murine MAP6 KO neurons, a net reduction in the numbers of intraluminal densities was observed. The presence of MAP6 inside MTs would explain, in part, the extremely slow turnover of MAP6 on neuronal microtubules described by Tortosa et al. (2017). Strikingly, intraluminal MAP6 induced MT coiling *in vitro*, leading to the formation of helical MTs with a highly conserved pitch and width associated with a specific tubulin compaction state in the MT lattice. This coiling pattern requires the Mn and Mc modules, as well as the first 35 N-terminal residues.

In neurons, the functions of such stable helical MAP6-containing MTs remains to be determined. By occupying a greater width, helical MTs could influence the spatial organization of the MT network, help determine neurite or axonal width, or confer resistance to compressive loads, such as those encountered during development. The unhabitual tubulin compaction state of helical MTs could also be a mean to specifically recruit a set of proteins such as molecular motors.

From an evolutionary point of view, it will be interesting to discover when did the ability of mammalian neuronal MAP6 to behave as a MIP emerge? In other words, does the MAP6 ancestor SAXO in *Trypanosoma* (Dacheux et al., 2012) behave as a MIP only, as a MAP only, or does it exhibit both properties?

### MAP6 Associates With Actin

The first indication that MAP6 can bind to actin was obtained when CaMKII-phosphorylated MAP6 was found to be unable to bind MTs, but that it binds actin *in vitro* and in the growth cones of neurons (Baratier et al., 2006). More recently, the central Mc modules of MAP6 were shown to bind actin and to induce specific rearrangements—leading to straightening and bundling of actin filaments (Peris et al., 2018). Moreover, MAP6-mediated stabilization of synaptic actin following synaptic activation was shown to be crucial for maintaining mature dendritic spines, the postsynaptic compartments of synapses (Peris et al., 2018). Other effects of MAP6 on the dynamic parameters of actin, such as nucleation, remain to be investigated, as does the possibility that MAP6 mediates cross-linking between actin and MT networks in neurons. MAP6 may also indirectly influence the actin cytoskeleton through an effect on signaling cascades as it has been shown to interact with the small GTPase protein Rac2 (Figure 1; Capala et al., 2015). Several MAP6 partners have also been shown to interact with actin (Supplementary Table 1), such as spinophilin (Grossman et al., 2004) or α-synuclein (Oliveira da Silva and Liz, 2020). These interactions support specific actin-related cellular functions for MAP6 and its partners.

### MAP6 Associates With Membranes and Neuroreceptors

#### MAP6 and Membranous Compartments

The N-terminal domains of MAP6 and MAP6d1 contain a stretch of cysteines with C5, C10, and C11 residues (Figure 2). These positions have been shown to be palmitoylated by a subset of palmitoylating enzymes containing a DHHC motif. The proteins are then targeted to the Golgi apparatus or the plasma membrane (Gory-Fauré et al., 2006, 2014). In addition, MAP6 interacts directly with ankyrin repeats present in the palmitoylating enzymes zDHHC13 and zDHHC17 (Lemonidis et al., 2015). During neuronal development, palmitoylated forms of MAP6 were identified on the Golgi and on secretory vesicles, and depalmitoylation by α/β hydrolase-domain-containing 17 proteins (ABHD17 proteins) induced MAP6 relocalization to MTs in the proximal part of the axon (Tortosa et al., 2017). In neurons, non-centrosomal MT nucleation is crucial, and through its interactions with MTs as well as with the Golgi apparatus or Golgi outposts which are nucleation sites for non-centrosomal MTs (Sanders and Kaverina, 2015; Valenzuela et al., 2020; Yang and Wildonger, 2020), MAP6 might contribute to such events.

MAP6 and MAP6d1 proteins were also shown to localize to mitochondria. This localization involved their N-terminal domain, but not palmitoylation (Gory-Fauré et al., 2014).

Finally, indirect evidence links MAP6 to lysosomes: KIF5B (Kinesin 1 heavy-chain) preferentially binds to MAP6-positive MTs (Tortosa et al., 2017) and lysosomal transport—which is dependent on KIF5B and on TMEM106B, a MAP6 partner (Figure 1)—is perturbed when MAP6 expression is impaired (Schwenk et al., 2014).

Overall, these results show that MAP6’s association with membranous compartments plays important roles in establishing and maintaining neuronal morphology. The several endocytosis-related proteins such as Varp, dynamin 1 or endophilin 1 (Craft et al., 2008; Vikhreva et al., 2009) which have been found to interact with MAP6 (Supplementary Table 1) suggests that the roles played by MAP6 in intracellular organelle trafficking still holds many secrets.

#### MAP6 and Calcium Channels

Proteomic analysis of the nano-environment of calcium channels (Ca_v_2.1, Ca_v_2.2, Ca_v_2.3, Ca_v_Beta3, Ca_v_Beta4) revealed the presence of MAP6 (Müller et al., 2010; Figure 1). More recently, MAP6, through its Mn3 module, was found to associate with Tctex1 (Brocard et al., 2017), a dynein light chain interacting with Ca_v_2.2/N-type calcium channels (Lai et al., 2005). In conjunction with Tctex1, MAP6 is involved in sorting and trafficking Ca_v_2.2 channels, as shown by impaired calcium signaling in MAP6 KO neurons (Brocard et al., 2017).

#### MAP6 and Neuroreceptors

Even if MTs are only transiently present in both pre- and post-synaptic compartments of synapses (axonal boutons and dendritic spines), MAP6 was consistently identified in synaptic proteomes, suggesting MT-independent roles (Peng et al., 2004; Baratier et al., 2006; Cheng et al., 2006; Collins et al., 2006; Munton et al., 2007; Weingarten et al., 2014). In a study related to subicular neurons from the hippocampus, MAP6 was associated with the receptors Neuropilin1, Plexin D1, and VEGFR2—which together make up the tripartite Semaphorin 3E receptor (Deloulme et al., 2015; Figure 1).

### MAP6 Is Involved in Signaling Cascades

In addition to its associations with subcellular compartments and receptors, MAP6 protein contains proline-rich domains (PRD), which are involved in the binding of SH3-domain-containing proteins (Figure 1 and Supplementary Table 1). Binding of Intersectin 1, cSrc, PLC-γ, or PI3K have all been described (Morderer et al., 2012; Deloulme et al., 2015). One of the PRD domains in MAP6 is essential to the Semaphorin 3E signaling cascade (Deloulme et al., 2015), driving the formation of the fornix, an axonal tract which requires Semaphorin 3E signaling.

Although some interactions with kinases and phosphatases have been reported (Figure 1), almost nothing is known about how MAP6’s functions are regulated by phosphorylation enzymes, with the exception of CaMKII. MAP6-N protein contains four CaMKII phosphorylation sites located within its calmodulin/MT-binding domains. These domains can be phosphorylated *in vitro* and *in vivo*. Phosphorylation induces MAP6 to detach from MTs and delocalize to actin within growth cones or dendritic spines (Baratier et al., 2006; Peris et al., 2018).

Finally, MAP6 was shown to associate with the highly brain-enriched BCH (Cdc42GAP Homology)-domain-containing protein Bmcc1/Prune2 (Figure 1) which negatively regulates the actin cytoskeleton regulator RhoA (Soh and Low, 2008). This association promotes the emergence of membrane protrusions (Arama et al., 2012). However, the relationship between a direct or indirect effect on the cytoskeleton is not clear.

### Physio-pathological Roles of MAP6 in the Central Nervous System

As neuronal MAPs, especially MAP6, were thought to be strong MT stabilizers, its deletion in mice was expected to be lethal due to major MT-breakdown in neurons. But in fact, MAP6 KO mice (also known as STOP KO mice) are viable, with an apparently normal brain organization (Andrieux et al., 2002). However, these mice display a wide range of biological and behavioral impairments reminiscent of symptoms displayed by patients suffering from psychiatric disorders, especially schizophrenia, as detailed below.

#### MAP6 KO Mice as a Model for the Study of Schizophrenia

MAP6 KO mice show hyperactivity, fragmentation of normal activity, anxiety-like behavior, social withdrawal, and impaired maternal behavior leading to the death of pups (Andrieux et al., 2002). These defects are associated with altered synapse-functioning, particularly during synaptic plasticity events when the synapses need to adapt their reactivity. These alterations lead, for example, to strong deficits in potentiation or depression of the synaptic responses. These biological and behavioral defects were shown to respond to long-term treatment with antipsychotics, the gold standard in schizophrenia treatment, thus positioning MAP6 KO mice as a useful model for the study of the physiopathology of this disease (Andrieux et al., 2002).

Schizophrenia is a chronic debilitating neurodevelopmental disorder that affects approximately 1% of the population worldwide; the first symptoms emerge during adolescence and early adulthood. It is characterized by a combination of positive (auditory and visual hallucination), negative (social withdrawal, anxiety), and cognitive symptoms (impaired memory, decision-making difficulties; Joseph et al., 2015). Following our seminal 2002 article (Andrieux et al., 2002), numerous studies were performed and their findings reinforced the validity of the MAP6 KO model for the study of schizophrenia. Indeed, MAP6 KO mice fulfill the three—construct/face/predictive—criteria for the validity of an animal model (Belzung and Lemoine, 2011; Jones et al., 2011) with similar underlying molecular defects/phenotypes/pharmacological responses, respectively (Delotterie et al., 2010; Volle et al., 2013; Deloulme et al., 2015; Bouet et al., 2021).

In further support of the validity of this model, subsequent studies revealed defects in MAP6 expression in humans presenting developmental delay, corpus callosum dysgenesis, autistic or schizophrenic symptoms (Shimizu et al., 2006; Choi et al., 2009; Martins-de-Souza et al., 2009; Xiao et al., 2015; Coumans et al., 2016; Wei et al., 2016a, b; Chen et al., 2021).

#### MAP6 Roles in Neurotransmission and Impact on Behavior

In tight correlation with the hyper-dopaminergia observed in schizophrenic patients (Carlsson et al., 2000; Kapur, 2003), MAP6 KO mice present increased dopamine overflow in the mesolimbic pathway. This system plays a significant role in mediating pleasure and rewarding experiences (Brun et al., 2005; Bouvrais-Veret et al., 2007, 2008). Schizophrenia also presents positive symptoms, and MAP6 KO mice exhibited increased locomotor activity (Andrieux et al., 2002; Brun et al., 2005; Fradley et al., 2005; Bouvrais-Veret et al., 2007) associated with hypersensitivity to novelty or to the psychostimulant amphetamine (Brun et al., 2005; Bégou et al., 2007; Bouvrais-Veret et al., 2008), along with extensive disruption of sleep patterns (Profitt et al., 2016). All these processes crucially depend on dopaminergic neurotransmission.

Negative symptoms in schizophrenia are mediated by alterations in both the glutamatergic and serotoninergic (5-HT) neurotransmission systems (Aghajanian and Marek, 2000; Carlsson et al., 2000). MAP6 KO mice exhibited abnormal glutamatergic neurotransmission with altered synaptic plasticity in the hippocampus, leading to totally defective Long-Term Potentiation (LTP) and Long-Term Depression (LTD), as measured by electrophysiology techniques (Andrieux et al., 2002). These defects are related to a smaller presynaptic vesicle pool (Andrieux et al., 2002), a decreased level of glutamate and the glutamate precursor glutamine in the forebrain (Brenner et al., 2007), as well as decreased mRNA levels of the glutamate transporter-1, Vglut1 (Eastwood et al., 2007). MAP6 KO mice are thus characterized by an overall hypo-glutamatergia.

If we now focus on serotoninergic neurotransmission, serotonin biosynthesis and expression of the serotonin (5-HT) receptors are highly perturbed in MAP6 KO mice, with a 70% increase in 5HT-1A expression in the raphe nuclei for example (Fournet et al., 2010), as well as half reduction of serotonin (5-HT) in the substantia nigra, the ventral tegmental area and the hippocampus (Fournet et al., 2012b). In addition, the levels of serotonin transporters (SERT) recapturing the serotonin (5-HT) released into the synaptic cleft is very severely affected in MAP6 KO mice with a decreased expression ranging from 30% to 90% in some brain areas whereas the expression increase up to 120% in other brain regions (Fournet et al., 2010). MAP6 KO mice thus display an extreme imbalance in serotoninergic transmission.

Finally, sensory-motor gating is also altered in MAP6 KO mice (Fradley et al., 2005; Volle et al., 2013). This effect might be linked to perturbed dopamine-, glutamate-, and serotonin-mediated neurotransmission, but could also be related to activation of opioid mu receptors (Quednow et al., 2008) which is altered in MAP6 KO mice (Charlet et al., 2010).

In summary, basal neurotransmission for all the major neurotransmitters is strongly perturbed in MAP6 KO mice. Strikingly, serotoninergic neurons—the longest and most extensively branched neurons in the brain—display very severe morphological defects, suggesting complementary roles of MAP6 in these neurons. These roles may include MT stabilization, modulation of serotonin neuroreceptors/transporters, and involvement in signaling cascades.

#### MAP6 Roles in Synaptic Plasticity

In terms of cognitive symptoms, MAP6 KO mice exhibit severe defects in both short-and long-term hippocampal synaptic plasticity. Indeed, glutamatergic hippocampal neurons in the CA1 region display severely defective Post-Tetanus Potentiation (PTP), and, as indicated above, cannot support LTP and LTD (Andrieux et al., 2002). Synaptic deficits exist in the presynaptic compartment, the axonal bouton, where a two-fold decrease in synaptic vesicles is reported (Andrieux et al., 2002) with a possible glutamate-release defect due to the absence of interaction between MAP6 and the SNARE protein SNAP25 (Figure 1). One can speculate on the formation of a transient complex between MAP6 and SNAP25 at the plasma membrane, in the presynaptic release zone, as the membrane targeting of both proteins is induced by similar palmitoylating enzymes (Greaves and Chamberlain, 2011; Gory-Fauré et al., 2014). In addition, the absence of MAP6 induces a general decrease in spine density which is related to altered actin dynamics in dendritic spines (Peris et al., 2018). MAP6 could also contribute to the entry of MTs into the synaptic compartments, and their residence time, thus influencing synaptic strength. Overall, these synaptic defects are most probably a consequence of MAP6’s involvement in signaling cascades, receptor homeostasis, and cytoskeleton regulation.

#### MAP6 Roles in Neuroanatomy

Although the first rough anatomical study of MAP6 KO mice revealed no obvious defects in brain anatomy (Andrieux et al., 2002), subsequent detailed studies showed that MAP6 KO mice have a reduced brain volume associated with an increased ventricular volume and a reduced cerebellum and thalamus size (Powell et al., 2007; Deloulme et al., 2015). Through the use of brain imaging techniques, MAP6 KO mice were found to exhibit a decrease in myelinated tract volumes (e.g., in the internal capsule and cerebellar peduncle) as well as a strong decrease in cortico-spinal tract fasciculation (Deloulme et al., 2015; Gimenez et al., 2017). A very intriguing structural defect is the absence of an important component of the limbic system in MAP6 KO mice—the post-commissural part of the fornix (Deloulme et al., 2015). The fornix is a tract that emerges from the subiculum, the most inferior component of the hippocampal formation of both hemispheres and extends to the mammillary bodies, within the hypothalamus. As part of the Papez circuit, the lack of the fornix leads to a dis-connectivity between the hippocampus and the hypothalamus and certainly contributes to the behavioral defects observed in MAP6 KO mice. During the formation of the fornix, Semaphorin 3E is an attractive guidance molecule for subicular neurons (Chauvet et al., 2007) as it binds to its tripartite receptor (Neuropilin 1/PlexinD1/VEGFR2) to induce activation of downstream signaling cascades involving SH3-containing proteins. Interestingly, the impaired fornix formation observed in MAP6 KO mice is not dependent on MAP6’s MT-binding domains; rather it is driven by the interaction of MAP6 PRD domains with key SH3-domain-containing proteins, including Intersectin 1, PI3K, and Src (Figure 1; Deloulme et al., 2015). The axonal tract defects identified in MAP6 KO mice correlate to a striking extent with anomalies identified on brain images from patients with schizophrenia (McCarley et al., 1999; De Peri et al., 2012; Shepherd et al., 2012; Bopp et al., 2017), including alteration of the fornix or of the cortico-spinal tract (Douaud et al., 2007; Kendi et al., 2008; Fitzsimmons et al., 2009; Luck et al., 2010; Davidson et al., 2012; Lee et al., 2013).

#### MAP6 Role in Neurogenesis

MAP6 is highly expressed in the olfactory bulb and the hippocampus (Couégnas et al., 2007; Richard et al., 2009), two regions where adult neurogenesis is known to occur. Several studies investigated adult neurogenesis in MAP6 KO mice. Benardais et al. (2010) reported an increased number of proliferating cells in the olfactory epithelium with increased apoptosis, whereas Fournet et al. (2010) discovered decreased cell proliferation in the hippocampus. At the molecular level, how MAP6 regulates neurogenesis remains a completely open question, although it is possible that MAP6 through its binding to the retinoic receptors RAR-beta and RAR-gamma (Figure 1) can modulate their functions known to be active during neurogenesis (Jacobs et al., 2006; Maden, 2007; Mishra et al., 2018).

#### MAP6 KO Mice Deficits and Therapeutic Approaches

As the defects observed in MAP6 KO mice are reminiscent of schizophrenia symptoms, the gold standard treatments for psychiatric diseases were the most obvious approach to try in an attempt to alleviate MAP6 KO deficiencies. Alternatively, new pharmacological approaches targeting the neuro-cytoskeleton represent promising avenues of investigation.

*Classical treatments of psychiatric diseases*. In accordance with their defects in almost all neuro-transmission systems, the defects observed in MAP6 KO mice are sensitive to neuroactive molecules. Thus, long-term treatments with both typical antipsychotics, such as haloperidol (Haldol) (dopamine antagonist), and atypical antipsychotics, such as risperidone (Risperdal) or clozapine (dopamine and serotonin antagonists), alleviate behavioral defects in MAP6 KO mice (Andrieux et al., 2002; Bégou et al., 2008; Delotterie et al., 2010). Moreover, clozapine improves alterations to synaptic plasticity (Delotterie et al., 2010) *via* its known positive effect on glutamatergic synapses (Fukuyama et al., 2019). Similarly, treatment of MAP6 KO mice with anti-depressants such as fluoxetine/Prozac (a Selective Serotonin Reuptake Inhibitor, gold standard treatment for depression) alleviated anxiety-like behavior and cognitive defects (Fournet et al., 2012a). The depressive-like behavior of MAP6 KO mice and their impaired hippocampal neurogenesis were alleviated by the use of electroconvulsive stimulation (ECS) (Jonckheere et al., 2018). ECS is the animal equivalent of Electroconvulsive Therapy (ECT), which remains a very powerful and useful treatment for major depression.

*Cytoskeleton-related drugs represent a new pharmacological approach to psychiatric disorders*. MAP6 KO mice were the first animal model to establish a link between cytoskeleton defects and the cognitive impairment characteristics of psychiatric disorders, in particular schizophrenia (Andrieux et al., 2002). Subsequently, other cytoskeletal components including actin and tubulin themselves, as well as CRMP1, CRMP2, MAP2, MAP1B, and LIM kinases were also linked to psychiatric disorders (see below and for review: Benitez-King et al., 2007; Gardiner et al., 2011; Zhao et al., 2015; Marchisella et al., 2016; Lasser et al., 2018). In addition, proteins that were initially identified as risk factors for mental illnesses, such as DISC1 (Disrupted In Schizophrenia) or dysbindin, were later shown to interact with MTs, MAPs, and actin (Morris et al., 2003; Hayashi et al., 2005; Talbot et al., 2006; Taya et al., 2007; Shimizu et al., 2008; Marley and von Zastrow, 2010; Bader et al., 2012).

In this context, cytoskeleton-related drugs have been investigated to determine their ability to influence biological and behavioral defects in MAP6 KO mice. Firstly, chronic exposure of MAP6 KO mice to Epothilone D (EpoD), a modulator of MT dynamics known to stabilize MTs *in vitro* (Chou et al., 1998; Kolman, 2004), leads to behavioral changes (improved maternal behavior, and concomitant pup survival), and improves short-term memory, associated with restoration of synaptic plasticity (LTP) in the hippocampus (Andrieux et al., 2006; Fournet et al., 2012a), as well as restoring neuronal transport deficits (Daoust et al., 2014). Second, a small peptide motif called NAP—present in Activity-Dependent Neuroprotective Protein (ADNP), which is dysregulated in schizophrenia and in autism (Gozes, 2011; Hacohen-Kleiman et al., 2018; Van Dijck et al., 2019)—partially alleviates cognitive impairments in MAP6 heterozygous mice (Merenlender-Wagner et al., 2010; Volle et al., 2013). NAP directly interacts with tubulin (Divinski et al., 2004) and also promotes MT growth and stability by its interaction with MT plus-end binding proteins of EB family (+TIPs proteins) (Gozes and Divinski, 2007; Oz et al., 2014). In addition, NAP has been shown to bind to Tau (Ivashko-Pachima et al., 2019) and to enhance its binding to MTs in cells (Ivashko-Pachima et al., 2017; Ivashko-Pachima and Gozes, 2019). Thus, NAP protective activity involves MT dynamics modulation *via* direct binding to tubulin and interaction with MT-associated proteins (Oz et al., 2014; Ivashko-Pachima et al., 2017). However, NAP’s effects in MAP6 KO mice might not be exclusively related to its MT-related properties as chronic NAP treatment restores normal levels of Beclin1 mRNA in MAP6-deficient mice (Merenlender-Wagner et al., 2014). Beclin1 is a key regulator of autophagy and its expression is strongly decreased in brains from patients with schizophrenia (Merenlender-Wagner et al., 2015).

Overall, these studies with EpoD and NAP highlight MAP6-mediated MT stabilization as an important feature for synaptic plasticity and behavior. The results presented also suggest that the cytoskeleton might be a relevant target for drug development to treat psychiatric disorders including schizophrenia.

#### Is MAP6 Present in Cilia and Linked to Schizophrenia Phenotypes?

MAP6 was the first neuronal MIP to be identified (Cuveillier et al., 2020). MIPs were originally described in the MT doublet of motile cilia and flagella axonemes (Kirima and Oiwa, 2018; Owa et al., 2019), where MTs needed to be highly stable to support the strong deformations required to produce a beating motion. The question of whether MAP6 also localizes to non-motile (primary) cilia or to motile cilia within the brain remains open. Future studies assessing these possibilities may provide additional molecular explanations for some of the cognitive impairments observed in MAP6 KO mice. Indeed, ciliopathies can lead to brain malformation and/or mental retardation (Reiter and Leroux, 2017). More precisely, primary cilia which act as signaling platforms participating in Sonic hedgehog or Wnt signaling pathways for example (Lee and Gleeson, 2010) have been shown to modulate neurotransmission through dopamine receptors expressed on their surface (Domire et al., 2011; Iwanaga et al., 2011; Leaf and Von Zastrow, 2015). Interestingly, Inversin and APT1—both MAP6 partners—have been shown to be part of the Wnt pathway (Supplementary Table 1). More directly, a reduced number of primary cilia was observed in the olfactory neuroepithelium in patients with schizophrenia (Muñoz-Estrada et al., 2018) and DISC1 was shown to be necessary for the formation of neuronal cilia (Marley and von Zastrow, 2010; Wang and Brandon, 2011). It would be interesting to investigate a possible role for MAP6 acting as a MIP in controlling the number and integrity of neuronal primary cilia.

## Crmps: from Signaling Pathways to Cytoskeleton Functions

Research on the structural MAPs revealed over time that their functions are not restricted to MT regulation. Simultaneously, other proteins that were first identified in signaling cascades, for example, were subsequently found to belong to the family of structural MAPs. Collapsin Response Mediator Proteins (CRMPs) are a perfect example of such proteins. Indeed, in addition to their well-known roles in signal transduction and neuronal physiology, we will summarize how, since the 1990s, CRMPs have been documented to play roles in regulating the cytoskeleton and especially in MT dynamics.

### CRMP Proteins as Signaling Proteins

Originally discovered in *C. elegans* and named ULIP (UNC-33 like phosphoprotein), members of the CRMP family are involved in neuronal connectivity for sensory and motor neurons (Brenner, 1974; Hedgecock et al., 1985). These proteins originated from various genes and were subsequently renamed Collapsin Response Mediator Proteins (CRMPs) due to their involvement in Semaphorin 3A guidance molecule signaling (Collapsin was the original name of Semaphorin 3A; Goshima et al., 1995).

CRMPs are substrates for a large number of kinases, and high levels of phosphorylation have been reported, mostly in the C-terminal domain (Cole et al., 2004, 2006; Zheng et al., 2018); CRMP phosphorylation has been extensively studied in the context of the axonal guidance signaling pathway induced by Semaphorin 3A. Several kinases involved in this signaling pathway phosphorylate CRMPs. First, Cdk5 acts as the priming kinase, phosphorylating CRMP1, 2, 4, and 5 in response to a Semaphorin 3A-signal *in vitro* and *in vivo* (Brown et al., 2004; Uchida et al., 2005). This phosphorylation is required for subsequent phosphorylation of CRMPs by GSK3β, shown to be a key factor in modulating CRMPs’ interaction with the cytoskeleton (Uchida et al., 2005; Yoshimura et al., 2005). Thus, the Semaphorin 3A-induced phosphorylation of CRMP2 by Cdk5 and GSK3β blocks its capacity to bind tubulin and MTs (Uchida et al., 2005; Yoshimura et al., 2005). This lack of interaction leads to cytoskeleton breakdown and the resulting collapse of the growth cone, as the functional consequences of Semaphorin 3A signaling.

Semaphorin 3A-stimulation of the growth of dendritic spines also involves CRMP1 phosphorylation by Cdk5, blocking its interaction with actin (Yamashita et al., 2011; Yao et al., 2016).

Phosphorylation events on CRMPs, thus regulate the proteins’ capacity to interact with the cytoskeleton while also modulating their interaction with other partners, such as RhoA (Alabed et al., 2007), the guidance cue co-receptor PlexinA1 (Deo et al., 2004), or the ion channels Cav2.2 (Brittain et al., 2009), and Nav1.7 (Dustrude et al., 2016).

At the same time, the C-terminal part of CRMP2 shares similarities with Tau PRD domains (Hensley and Kursula, 2016), opening up the possibility that CRMPs can bind to SH3-domain-containing proteins.

### CRMPs Bind and Stabilize Microtubules

From the time of their discovery in 1985, CRMPs were linked to MTs since mutations induced an over-abundance of MTs in axonal shafts (Hedgecock et al., 1985). It was therefore proposed that CRMP could modulate axonal outgrowth by stabilizing the cytoskeleton (Hedgecock et al., 1985). More recent work has indicated that all CRMPs bind tubulin both *in vitro* and *in vivo* (Fukata et al., 2002a; Lin et al., 2011; Khazaei et al., 2014), although the interaction between CRMP2 and MTs has been the focus of this particular study. Indeed, for CRMP2, its interaction with MTs was recently found to involve two distinct domains: the N-terminal domain, which is essential for binding to soluble tubulin, thus promoting MT polymerization; and the C-terminal region, which interacts with and stabilizes polymerized microtubules (Niwa et al., 2017). Although the ability to bind MTs is shared by all CRMPs, the MT-stabilizing capacity reported for CRMP2 is only shared by CRMP1 and CRMP4 (Lin et al., 2011). In contrast, CRMP5 does not influence MT dynamics (Brot et al., 2010), and CRMP3 was shown to inhibit MT polymerization (Aylsworth et al., 2009). The role of CRMP2 in MT assembly was found to be crucial for neurite formation and axon development (Fukata et al., 2002b) whereas CRMP4-dependent MT organizations contribute to growth-cone dynamics (progression, pausing, and retraction) as well as to axon elongation and regeneration (Khazaei et al., 2014).

### CRMPs Associate With Actin

Of all the isoforms, only CRMP3 presents weaker or no interaction with actin (Tan et al., 2015). All other CRMPs have an actin-binding site located at their C-terminal end, adjoining their MT-binding site. This actin-binding site was initially characterized in CRMP4 and promotes the formation of F-actin both *in vitro* and *in vivo* (Rosslenbroich et al., 2005; Khazaei et al., 2014; Cha et al., 2016). In neurons, through their action on the actin cytoskeleton, CRMP4 and CRMP5 contribute to neurite outgrowth and growth-cone remodeling (Khazaei et al., 2014; Gong et al., 2016), and CRMP4’s actin-binding capacity also contributes to dendrite maturation in hippocampal neurons (Cha et al., 2016). The effects of CRMP1 and CRMP2 on actin dynamics are more indirect, as interactions with VASP family proteins or the Sra-1 / WAVE1 complex are required for these proteins to influence axon formation and growth (Kawano et al., 2005; Yu-Kemp and Brieher, 2016; Yu-Kemp et al., 2017).

### CRMPs Associate With Membranes and Neuroreceptors

The role of CRMPs in vesicular trafficking and plasma membrane targeting of several transmembrane proteins is well-documented. Thus, CRMP2 is known to bind to the vesicle-associated proteins Slp1 and Rab27 allowing anterograde transport of BDNF receptor TrkB (Arimura et al., 2009). It also binds to the endocytic adaptor Eps15, the ubiquitin ligase Nedd4.2, and to α-adaptin which regulates clathrin-dependent endocytosis of the cellular adhesion molecule L1 and the sodium channel Nav1.7 (Nishimura et al., 2003; Kawano et al., 2005; Dustrude et al., 2016). CRMP1 modulates Na^+^ currents by interacting with Nav1.7 (Yamane et al., 2017) whereas, like MAP6, CRMP2, and CRMP3 respectively interact with voltage-gated Cav2.2/N-type and L-type channels (Brittain et al., 2009; Chi et al., 2009; Quach et al., 2013). CRMP4 is involved in vesicular trafficking through mechanisms involving binding to the SH3 domains of the scaffolding protein Intersectin 1 (Quinn et al., 2003)—which is also a MAP6 partner (Figure 1).

Despite the early discovery of the involvement of CRMPs in the Semaphorin 3A signaling pathway (Goshima et al., 1995), little is known about direct interactions between CRMPs and the numerous semaphorin receptors represented by the Neuropilin and Plexin families. CRMP1, 2, 3, and 4 complexes with Plexin-A1 have been reported following over-expression of the different isoforms in the COS7 cell line (Deo et al., 2004), and associations with the mono-oxygenases MICALs have also been described (Schmidt et al., 2008). In addition, CRMP2 was shown to interact with Plexin-A2 and A3 in the Nogo and Semaphorin 3A signaling pathways, respectively (Sekine et al., 2019; Jiang et al., 2020).

### Physio-pathological Roles of CRMPs in the Central Nervous System

As part of their regulation of neuronal development and plasticity, CRMPs are involved in many neurodevelopmental processes including neural progenitor proliferation (Charrier et al., 2006), neuronal migration (Yamashita et al., 2006), and neuronal morphogenesis with both axonal and dendritic maturation influences. The study of CRMP1, CRMP2, and CRMP4 KO neurons revealed morphological defects, especially in dendritic development and branching, but also in migration and positioning (Yamashita et al., 2006, 2007; Niisato et al., 2012). These phenotypes are more severe in double KO neurons, whether the combination is CRMP1 and CRMP4, or CRMP2 and CRMP4 (Tan et al., 2015; Yamazaki et al., 2020). *In vivo*, KO of any CRMP (CRMP1, CRMP2, CRMP3, or CRMP4) leads to robust alteration of dendritic morphogenesis in several brain regions including the hippocampus and the cortex (Yamashita et al., 2007; Quach et al., 2008; Niisato et al., 2012, 2013; Yamashita and Goshima, 2012; Tsutiya et al., 2015, 2016). CRMP2 is also strongly associated with axonal specification (Nishimura et al., 2003; Kawano et al., 2005; Yoshimura et al., 2005; Morita and Sobue, 2009). The extensive effects of CRMPs on neuronal differentiation are stronger than those observed for the deletion of classical MAPs. This enhanced effect might be due to the crucial roles of CRMPs at the interface between elements of the cytoskeleton and signaling proteins. Impairment of CRMPs functions also leads to synaptic-plasticity defects with abnormal LTP (Su et al., 2007; Quach et al., 2008, 2011; Yamashita et al., 2011), linked to impaired learning and memory (Su et al., 2007; Yamashita et al., 2013).

In humans, CRMPs are associated with several psychiatric disorders, with striking numbers of publications providing evidence of links between CRMPs and schizophrenia (Nakata et al., 2003; Nakata and Ujike, 2004; Ujike et al., 2006; Koide et al., 2010; Hensley et al., 2011; Bader et al., 2012; Lee et al., 2015; Quach et al., 2015; Toyoshima et al., 2019). For example, polymorphisms in the genes encoding CRMP1 and CRMP2, as well as altered hippocampal expression of CRMP2 and CRMP4 have been reported in patients with schizophrenia (Edgar et al., 2000; Beasley et al., 2006; Föcking et al., 2011; Bader et al., 2012). In addition, the abnormal sensory-motor gating abilities reported in psychiatric patients and described for MAP6 KO mice (Fradley et al., 2005), were also replicated in CRMP1 and CRMP3 KO mice (Quach et al., 2008; Yamashita et al., 2013). Importantly, these defects could be alleviated through the use of the antipsychotic chlorpromazine (Yamashita et al., 2013), and CRMP2 and CRMP4 phosphorylation states were shown to be downregulated by the antipsychotics Clozapine and Risperidone (Kedracka-Krok et al., 2015).

### Which Proteins Can Be Classified as MAPs?

In summary, the ability of CRMPs to bind MTs, and thus promote their polymerization and stabilization, are major arguments to consider them as members of the wider MAP family. Moreover, and as indicated above, like other MAPs, CRMPs can also bind actin and are involved in signaling cascades. As for conventional MAPs, deletion of CRMPs in mice does not lead to severe cytoskeleton alterations, but rather to subtler neurodevelopmental defects and cognitive dysfunctions similar to those encountered in psychiatric diseases. The history of protein identification draws attention toward specific research. In the case of CRMPs, although data regarding their interactions with MTs have been produced they have not been extensively investigated in the field of cytoskeleton research. In particular, they have never been used in *in vitro* cell-free systems. Several basic questions thus remain open, such as: Do CRMPs modulate specific parameters of microtubule dynamics? Do they induce MT bundling or simply MT polymerization?

Our demonstration that CRMPs can be considered as MAPs could be extended to other proteins like for example, the Huntingtin protein or α-synuclein. These two proteins have been actively studied in the context of neurodegenerative diseases. Indeed, HTT binds to MTs and actin, is involved in signaling cascades, is palmitoylated by the same DHCC as MAP6 (Lemonidis et al., 2015), and interacts with neuroreceptors (for review see Saudou and Humbert, 2016). Similarly, α-synuclein, a MAP6 partner (Figure 1), interacts with and nucleates MTs (Cartelli et al., 2016). α-synuclein also interacts with actin and reduces F-actin polymerization speed (Sousa et al., 2009; Cartelli et al., 2016; Oliveira da Silva and Liz, 2020). It also participates in signaling cascades and neuroreceptor functions (for review see Emamzadeh, 2016; Bernal-Conde et al., 2019). In addition to these examples, proteins related to schizophrenia susceptibility could be mentioned, such as DISC1 (Disrupted In Schizophrenia) or dysbindin, both of which have been shown to interact with MTs, MAPs, and actin (Morris et al., 2003; Hayashi et al., 2005; Talbot et al., 2006; Taya et al., 2007; Shimizu et al., 2008; Marley and von Zastrow, 2010; Bader et al., 2012).

## Common Properties of The Neuronal Structural MAPs

In this section, we will briefly summarize the properties of the classical neuronal structural MAPs (Tau, MAP1, and MAP2) and compare them to those described for MAP6 and CRMPs. We will focus particularly on properties that are just coming to light.

All the classical MAPs bind to MTs and induce various levels of stability (Baas et al., 1994; Bulinski and Bossler, 1994; Vandecandelaere et al., 1996; Faller et al., 2009; Kadavath et al., 2015; Qiang et al., 2018). Recent advances in cryo-EM have made it possible to visualize how Tau binds along protofilaments at the interface between tubulin dimers (Kellogg et al., 2018). Elucidating the near-atomic structure of complexes between the other MAPs and MTs is essential if we wish to reveal the common and specific mechanisms behind MT stabilization for each MAP. Interestingly, several classical MAPs such as MAP1B, MAP2, and Tau, are also able to indirectly modulate MT dynamics *via* their interaction with EB proteins (Kapitein et al., 2011; Tortosa et al., 2013; Sayas et al., 2015; Ramirez-Rios et al., 2016). Such a possibility regarding MAP6 and CRMPs has not yet been investigated and could be of interest.

With regard to the ability of MAP6 to behave as a MIP, future studies will reveal whether this property is shared by other structural MAPs. Although some previous works suggested that Tau binds to the luminal side of MTs (Kar et al., 2003; Inaba et al., 2019), the answer to this question remains elusive. Interestingly, actin was very recently discovered inside the MT lumen (Paul et al., 2020), opening the possibility that MAPs/MIPs might be involved in the crosstalk between the two cytoskeletons in the MT lumen like they are in the cytoplasm.

Like MAP6, the other structural MAPs—Tau, MAP1, and MAP2—were shown to bind actin (Griffith and Pollard, 1982; Pedrotti et al., 1994; Ozer and Halpain, 2000; Roger et al., 2004; Ding et al., 2006) and to regulate synaptic plasticity through actin-dependent mechanisms (Davidkova and Carroll, 2007; Tortosa et al., 2011; Benoist et al., 2013; Takei et al., 2015). Interestingly, Tau was directly shown to co-organize actin and MT networks *in vitro* and in the neuronal growth cone (Elie et al., 2015; Biswas and Kalil, 2018), this ability may be shared by the other MAPs (Mohan and John, 2015).

In relation to neuroreceptors, like MAP6 (Figure 1), MAP1 and MAP2 proteins interact with the Cav2.2/N-type calcium channel or with BKCa potassium channels (Davare et al., 1999; Park et al., 2004; Leenders et al., 2008). In terms of membrane-association, Tau and MAP2 interact with the membranes of the Golgi and the endoplasmic reticulum (Farah et al., 2005, 2006), whereas MAP1S was shown to link mitochondria and autophagosomes to MTs (Xie et al., 2011).

Several other MAPs in addition to MAP6 contain PRD domains and thus interact with SH3-containing proteins. For example, Tau and MAP2 bind to various SH3-containing proteins including the non-receptor tyrosine kinase Src (MAP6 partner, Figure 1; Lim and Halpain, 2000). Interestingly, in link with Alzheimer’s disease, a general inhibition of Tau’s interactions with SH3-domain-containing proteins was shown to reduce Amyloid β-induced membrane trafficking abnormalities and neurite degeneration (Rush et al., 2020).

Finally, as with MAP6 deletion, invalidation of other MAPs in mice did not result in major MT-breakdown or in lethal neurodevelopmental defects but rather produced viable mice (Harada et al., 1994; Takei et al., 1997, 2015; Teng et al., 2001). However, all KO mice display cognitive dysfunctions similar to those associated with psychiatric diseases. Thus, Tau KO mice exhibit impaired neurogenesis (Dioli et al., 2017), hippocampal synaptic plasticity, and cognitive defects (Ikegami et al., 2000; Lei et al., 2012; Ahmed et al., 2014; Ma et al., 2014; Regan et al., 2015), as well as age-dependent brain atrophy associated with loss of neurons and synapses (Lei et al., 2012). MAP1B-deficient mice also display abnormal synapse maturation (Tortosa et al., 2011; Bodaleo et al., 2016), along with synaptic transmission defects due to deregulation of AMPA receptor trafficking (Benoist et al., 2013; Palenzuela et al., 2017), and impaired synaptic plasticity with abnormal LTP (Zervas et al., 2005). Importantly, MAP1B has been linked to the protein KIRREL3 which is associated with autism/intellectual disability (Liu et al., 2015). This protein is associated with the altered working memory observed in young people with attention-deficit/hyperactivity disorder (Salatino-Oliveira et al., 2016). Mutated forms of MAP1B have been linked to intellectual disability and extensive white-matter deficits in humans (Walters et al., 2018).

A review of the literature relating to MAPs, in particular, Tau (which is by far the most extensively studied MAP), reveals that some MAP features have not yet been reported for MAP6 and CRMPs. Firstly, it is quite clear that Tau is a *bona fide* nuclear protein (for review see Bukar Maina et al., 2016) mainly contributing to defending the genome against cellular stress. As shown in Supplementary Table 1, MAP6 has been shown to interact with various nuclear receptors and effectors, but nothing is known about its possible roles in the nucleus. Secondly, Tau is an unstructured protein that has been shown to promote phase-separation events in cells, both as part of physiological processes such as axonal transport and in pathological conditions such as during Tau aggregation (Hernández-Vega et al., 2017; Wegmann et al., 2018; Siahaan et al., 2019). How the capacity of Tau to promote phase separation affects its non-cytoskeleton-related functions still remains to be determined. As most MAPs including MAP6 are unstructured proteins, it appears logical that the ability to contribute to phase separation events will be shared by many MAPs.

## Conclusion

Structural MAPs, presented here through the examples of MAP6 and CRMPs, are highly versatile proteins with multiple partners and functions, playing major roles in several brain functions.

The original classification of MAPs was based on their ability to bind MTs. This binding may contribute to MT stability, but it might also be crucial to ensure MAPs presence all over the cell in order to be available to promote signal propagation and/or to form multi-protein complexes (post-synaptic densities) or regulate the protein composition of membrane compartments. In other words, the MT-binding ability of MAPs is probably required for all their other functions as it is essential to ensure specific spatial and temporal localization. These dual abilities of MAPs to stabilize MTs and to use them as a means to gain access to all regions of the cell for other functions makes it experimentally impossible to distinguish between their MT-related and -unrelated functions.

## Author Contributions

CC, BB, CR, ED, J-CD, SG-F, CD, CB, IA and AA wrote the manuscript. All authors contributed to the article and approved the submitted version.

## Conflict of Interest

The authors declare that the research was conducted in the absence of any commercial or financial relationships that could be construed as a potential conflict of interest.
