# Supplementary material for: Beyond Neuronal Microtubule Stabilization: MAP6 and CRMPS, Two Converging Stories
Source: Front Mol Neurosci. 2021 May 5;14:665693. doi: 10.3389/fnmol.2021.665693 (PMC8131560; doi:10.3389/fnmol.2021.665693)
Supplement: Supplementary file 1 [file Table_1.pdf]

Supplementary Table 1: MAP6 partners								
**Category**	**Partner**	**Human gene**	**Name and aliases**	**Localizations and Roles**	**MAP6 partners found in the experiment**	**Identification methods**	**Ref**	**Confidence**
**Cytoskeleton**	**Microtubules**	Multiple genes	α-Tubulin / β-Tubulin	-Ubiquitous, cell body, dendrites, axon<br>-Vesicular transport, neuronal polarization, synaptic plasticity		-Co-pelleting<br>-IF<br>-NMR	(1-3)	Strong
	**Actin**	Multiple genes	F-Actin	-Ubiquitous, cell body, dendrites, axon<br>-Cell motility, structure and plasticity		-Co-pelleting<br>-IF<br>-FRET	(4, 5)	Strong
	**Neurofilament**	Multiple genes	Neurofilament light, medium and heavy chains	-Neuronal, enriched in axons<br>-Neuronal caliber, intracellular transport<br>-Form spheroid aggregates in Amyotrophic Lateral Sclerosis		-Co-purification	(6)	Moderate
	**Inversin**	INVS	Inversin/<br>NPHP2 / INV / NPH2	-Ubiquitous, basal body of the cilia<br>-Wnt signaling pathway<br>-Nephronophthisis (ciliopathy)	Tubulin<br>Actin<br>14-3-3 ζ/δ	-Peptide-affinity column	(9)	Moderate
	**Bmcc1s**	PRUNE2	Prune homolog 2<br>BMCC1 / BNIPXL / Prune2	-Enriched in the brain<br>-Bmcc1s variant bound to microtubules<br>-Proapoptotic<br>-Potential biomarker of AD	Tubulin	-GST pull down<br>-Co-IP	(10)	Strong

**Cytoskeleton**	**Spinophilin**	PPP1R9B	Protein Phosphatase 1 Regulatory subunit 9B / Spn / SPINO / PPP1R6 / Neurabin-2	-Ubiquitous, cell body, dendrites, axon, dendritic spines<br>-Scaffold between membranes and cytoskeleton<br>-Spine morphology, synaptic plasticity, neuronal migration<br>-Decreased mRNA in MAP6 KO mice (11)<br>-Psychiatric disorders	Tubulin/Actin<br>Neurofilament<br>Dynamin 1<br>CamKII<br>14-3-3 ζ/δ<br>α-Adaptin<br>Endophilin 1<br>SNAP25<br>Agap2<br>Calmodulin<br>MAP6D1	-Co-IP	(12)	Moderate
	**Tctex1**	DYNLT1	T-complex testis expressed 1 / Dynein light chain Tctex-type 1	-Ubiquitous, cell body, dendrites, axon<br>-Surface expression of Ca_v<br>-Ca^{2+} influx<br>-Actin remodeling in neurite<br>-Primary cilium length		-Yeast 2 Hybrid<br>-Co-IP	(8)	Strong
	**MAP6d1**	MAP6D1	MAP6 domain containing protein 1 / SL21 / STOP-like protein of 21 kDa	-Strictly neuronal<br>-Golgi apparatus<br>-Cytoskeleton		-Yeast 2 Hybrid<br>-Co-IP	(13)	Strong
	**MAP6**	MAP6	Microtubule-Associated Protein 6 / Stable Tubule Only Polypeptide (STOP)			-Yeast 2 Hybrid<br>-Co-IP	(13)	Strong
**Endocytosis**	**SNAP25**	SNAP25	Synaptosome-associated protein 25 / SEC9	-Pre-synaptic compartment<br>-t-SNARE vesicle fusion<br>-Calcium regulated	Tubulin	-Co-IP	(14)	High

**Endocytosis**	**Intersectin 1**	ITSN1	Intersectin-1 / ITSN / SH3D1A / SH3P1	-Pre and post-synaptic compartments<br>-Adaptor protein for Clathrin-Mediated Endocytosis<br>-GEF for CDC42<br>-Actin remodeling (N-WASP activation)	Dynamin 1	-GST pull-down<br>-Co-IP	(7, 15)	Strong
	**Dynamin 1**<br>**Endophilin 1**<br>**α-Adaptin**	DNM1	Dynamin-1 / DNM / EIEE31	-Pre and post-synaptic compartments<br>-Vesicle neck constriction<br>-Vesicle transport and targeting<br>-Actin remodeling	BK_{Ca}	-GST pull-down with a mix of the 3 proteins	(16)	Moderate
		SH3GL2	Endophilin A1 / SH3 domain containing GRB2like 2 / CNSA2 / SH3P4 / EEN-B1 / SH3D2A	-Pre and post-synaptic compartments<br>-Vesicle endocytosis<br>-Dendritic spine morphogenesis and plasticity				
		AP2A2	α-Adaptin C / Adaptor-related protein complex 2 subunit α-2 / ADTAB / CLAPA2	-Pre and post-synaptic compartments<br>-Vesicle recycling via Clathrin-Mediated Endocytosis				
	**TMEM106B**	TMEM106B	Transmembrane protein 106B / HLD16	-Membrane of lysosomes<br>-Lysosomal function<br>-Frontotemporal lobar degeneration		-Co-IP	(17)	Strong
	**Varp**	ANKRD27	Ankyrin repeat domain 27 / PP12899	-Ubiquitous, cell body, dendrites, axon<br>-Early endosomes<br>-GEF for Rab21<br>-Neurite growth		-GST pull-down	(18)	High

**Endocytosis**	**Bri2**	ITM2B	Integral membrane protein 2B / BRI / FBD / ABRI / E25B / E3-16 / RDGCA / BRICD2B	-Ubiquitous, cell body, dendrites, axon<br>-Transmembrane protein in ER, Golgi apparatus, plasma membrane and vesicles<br>-Regulation of Aβ oligomerization levels<br>-Neuron maturation<br>-Familial British and Danish dementia	Agap2<br>Neurofilament<br>SNAP25<br>Dynamin 1<br>CaMKII<br>Endophilin 1<br>Tubulin	-Co-IP	(19)	Moderate
	**α-Synuclein**	SNCA	α-Synuclein / PD1 / NACP / PARK1 / PARK4	-Pre-synaptic compartment<br>-Synaptic vesicles release<br>-SNARE protein folding	Neurofilament<br>CAMKII<br>Actin<br>Tubulin<br>14-3-3 ζ/δ	-Peptide-affinity column	(20)	Moderate
**Neuro-receptors & channels**	**Ca_v2.1**	CACNA1A	Calcium voltage-gated channel subunit α-1 A / CACNL1A4 / EA2 / FHM / MHP / APCA / HPCA / SCA6	-Pre-synaptic compartment<br>-Fast synaptic transmission<br>-Familial Hemiplegic migraine and Episodic ataxia 2<br>-Spinocerebellar ataxia 6	CAMKII<br>Intersectin 1<br>BK_{Ca}<br>SNAP25<br>Tubulin	-Co-IP	(21)	Moderate
	**Ca_v2.2**	CACNA1B	Calcium voltage-gated channel subunit α-1 B / CACNN / DYT23 / NEDNEH / CACNL1A5	-Pre-synaptic compartment<br>-Neurotransmitter release<br>-Fast synaptic transmission	CAMKII<br>14-3-3 ε<br>Intersectin 1<br>BK_{Ca}<br>SNAP25<br>Tubulin	-Co-IP	(8, 21)	Strong
	**Ca_v2.3**	CACNA1E	Calcium voltage-gated channel subunit α-1 E	-Pre-synaptic compartment<br>-Modulation of neuronal firing	SNAP25<br>Tubulin	-Co-IP	(21)	Moderate
	**Ca_vBeta3**	CACNB3	Voltage-dependent L-type calcium channel subunit beta-3 / CAB3 / CACNLB3	-Pre- and post-synaptic compartments<br>-L-type calcium channel subunit	CAMKII<br>Ca_v2.1<br>Ca_v2.2<br>Ca_v2.3<br>SNAP25			

**Neuro-receptors & channels**	**Ca_vBeta4**	CACNB4	Calcium voltage-gated channel auxiliary subunit beta 4 / EA5 / EJM / CAB4 / EIG9 / EJM4 / EJM6 / CACNLB4	-Pre- and post-synaptic compartments<br>-Possible component of P-/Q-type calcium channel	CAMKII<br>Ca_v2.1<br>Ca_v2.2<br>Ca_v2.3<br>SNAP25			
	**BK_{Ca}**	KCNA1	Potassium voltage-gated channel subfamily A member 1 / EA1 / MK1 / AEMK / HBK1 / HUK1 / MBK1 / RBK1 / KV1.1	-Brain, plasma membrane<br>-Tetrameric voltage-gated delayed potassium channel<br>-Myokymia with periodic ataxia	Tubulin	-GST pull-down	(22)	Moderate
	**Plexin D1**	PLXND1	Plexin-D1 / PLEXD1	-Cortical, subicular and striatal neurons<br>-Guidance receptor of Sema3E		-Co-IP	(7)	Moderate
	**Neuropilin 1**	NRP1	Neuropilin 1 / NP1 / NRP / BDCA4 / CD304 / VEGF165R	-Neurons<br>-Receptor of several class 3 semaphorin<br>-Sema3E attractive pathway<br>-Co-receptor of PlexinD1				
	**VEGFR2**	KDR	Kinase insert domain receptor / FLK1 / CD309 / VEGFR	-Neurons and astrocytes<br>-Receptor tyrosine kinase<br>-Neurogenesis, neuronal survival, synaptic plasticity<br>-Sema3E attractive pathway, co-receptor of plexin D1				

**Signal transduction**	**zDHHC13**	ZDHHC13	Zinc finger DHHC-type palmitoyltransferase 13	-Wide cellular expression<br>-Golgi apparatus and vesicles<br>-Palmitoyltransferase	SNAP25	-Yeast 2 Hybrid	(23)	Moderate
	**zDHHC17**	ZDHHC17	Zinc finger DHHC-type palmitoyltransferase 17 / HIP14	-Golgi apparatus and vesicles<br>-Palmitoyltransferase specific of a subset of neuronal proteins (SNAP25, HTT, PSD95)		-Yeast 2 Hybrid<br>-His pull-down		Strong
	**ABHD17A**	ABHD17 A	Depalmitoylase α/β hydrolase domain-containing protein 17A / FAM108A1	-Plasma membrane, Golgi, vesicles<br>-Depalmitoylase		-Over-expression (induces delocalization of MAP6 from vesicles)	(24)	Moderate
	**ABHD17B**	ABHD17 B	Depalmitoylase α/β hydrolase domain-containing protein 17B / FAM108B1	-Plasma membrane, Golgi, vesicles<br>-Depalmitoylase				
	**ABHD17C**	ABHD17 C	Depalmitoylase α/β hydrolase domain-containing protein 17C / FAM108C1	-Plasma membrane, Golgi, vesicles<br>-Depalmitoylase				
	**APT1**	LYPLA1	Acyl-protein-thioesterase 1 / APT-1 / hAPT1 / Lysophospholipase A1 / LPL-1 / LysoPLA1	-Wide expression, cytosolic<br>-Depalmitoylase<br>-Dendritic spine morphogenesis<br>-Notch and Wnt signaling		-Multiplexed mass spectrometry<br>-Palmitoylation assay	(25, 26)	High
	**Calmodulin**	CALM1	CaM / CAM2 / CAM3 / CAMB / CAMC / CAMI / PHKD / CPVT4 / DD132 / CALML2 / CAMIII	-Ubiquitous<br>-Ca^{2+} signaling<br>-Synaptic transmission and plasticity	Actin<br>14-3-3<br>CaMKII<br>Endophilin 1<br>Tubulin<br>CAMKII	-Affinity-column<br>-Peptide spots<br>-Sepharose-affinity column<br>-GST pull down	(1, 27, 28)	Strong

**Signal transduction**	**Rac1**	RAC1	Rac family small GTPase 1 / MIG5 / MRD48 / TC-25 / p21-Rac1	-Plasma membrane-bound small GTPase<br>-Cytoskeletal rearrangement<br>-Axon growth and guidance		-Avi-tag Pull-down	(29)	Moderate
	**Rac2**	RAC2	Rac family small GTPase 2 / Gx / EN-7 / IMD73A / IMD73B / IMD73C / HSPC022 / p21-Rac2	-Plasma membrane-bound small GTPase<br>-Cytoskeletal rearrangement				
	**14-3-3 ζ/δ**	YWHAZ	Tyrosine 3-monooxygenase / tryptophan 5-monooxygenase activation protein zeta / HEL4 /KCIP-1 /HEL-S-3 / HEL-S-93 / POPCHAS	-Ubiquitous, cytosolic<br>-Binding to phosphoserine-containing proteins<br>-Cell signaling<br>-Neurogenesis<br>-Neurodevelopmental defects<br>-Neuropsychiatric disorders	Actin<br>CaMKII<br>Calmodulin<br>Neurofilament<br>Spinophilin<br>Tubulin	-Tandem affinity purification	(30)	Moderate
	**CAMKII**	Multiple genes	Calcium/calmodulin-dependent protein kinase II	-Post-synaptic compartment<br>-Serine/threonine kinase<br>-Dendritic spine and synapse formation<br>-Synaptic plasticity<br>-Autosomal Dominant Mental Retardation		-CaMKII-phospho sites (S198 and S491)	(5)	Strong
	**Agap2**	AGAP2	ArfGAP with GTPase domain / ankyrin repeat and PH domain 2	-Cytosolic, nuclear<br>-GTPase<br>-Anti-apoptotic (NGF, phosphoinositide 3-kinase)	PI3K	-Co-IP	(31)	Moderate
	**Src**	SRC	SRC proto-oncogene, non-receptor tyrosine kinase / ASV / SRC1 / THC6 / c-SRC / p60-Sr	-Cell body, dendrites, axon<br>-Non-receptor tyrosine kinase<br>-NMDA receptor activation		-GST pull-down	(7)	Moderate

**Signal transduction**	**p85/PI3K**	PIK3R1	Phosphoinositide-3-kinase regulatory subunit 1 / p85 / AGM7 / GRB1 / IMD36 / p85-ALPHA	-Ubiquitous, cytosolic<br>-Subunit of PI3K<br>-AKT/mTOR signaling pathway		-GST pull-down	(7)	Moderate
	**TNK2**	TNK2	Tyrosine kinase non-receptor 2 / ACK / ACK1 / p21cdc42Hs	-Cytosolic<br>-Non-receptor tyrosine kinase<br>-EGF signaling pathway<br>-Severe autosomal recessive infantile-onset epilepsy	Actin<br>Neurofilament<br>CaMKII<br>Tubulin<br>α-Adaptin	-Immuno-affinity column	(32)	High
	**Presenilin 1**	PSEN1	Presenilin-1 / AD3 / FAD / PS1 / S182 / ACNINV3	-Plasma membrane, endosomes<br>-Catalytic subunit of the γ-secretase complex<br>-Alzheimer Disease	Actin<br>CaMKII<br>Tubulin	-GST pull down	(33)	Moderate
**Nuclear receptors & effectors**	**Coup-TF1**	NR2F1	Nuclear Receptor subfamily 2 group F member 1 / EAR-3 / SVP44 / BBSOAS / ERBAL3	-Nuclear, transcription factor<br>-Neuronal differentiation and development<br>-Bosch-Boonstra optic atrophy syndrome		-Yeast 2 Hybrid	(34)	High
	**RAR-beta**	RARB	Retinoic Acid Receptor beta / HAP / RRB2 / NR1B2 / MCOPS12	-Nuclear<br>-Neuronal differentiation<br>-Intellectual disability<br>-Motor impairment				High
	**RAR-gamma**	RARG	Retinoic Acid Receptor Gamma / RARC / NR1B3	-Nuclear<br>-Neuronal differentiation				High
	**RORB**	RORB	Retinoic Orphan Acid Receptor β / EIG15 / NR1F2 /RZR-BETA / bA133M9.1	-Nuclear<br>-Neurogenesis, axon growth<br>-Genetic Generalized Epilepsy				Moderate

	**SLX4IP**	SLX4IP	SLX4 interacting protein / C20orf94 / bA204H22.1 / bA254M13.1 / dJ1099D15.3	-Nuclear<br>-DNA maintenance and repair	14-3-3 ζ/δ<br>Actin<br>Tubulin	-Tandem affinity purification	(35)	Moderate
	**TIA1**	TIA1	T cell intracellular antigen / TIA1 cytotoxic granule associated RNA-binding protein / WDM / ALS26	-Nuclear, cytoplasmic stress granules<br>-Prion-related RNA-binding protein<br>-mRNA splicing<br>-Welander distal myopathy	α-Adaptin<br>SNAP25	-Immuno-affinity column	(36)	Moderate
	**HERC2**	HERC2	HECT and RLD domain containing E3 ubiquitin protein ligase 2 / D15F37S1 / jdf2 / SHEP1 / p528	-Nuclear, cytoplasmic<br>-E3 ubiquitin ligase<br>-Mental retardation, autosomal recessive 38 (MRT38)		-Co-IP<br>-Proteomic<br>-Bioinformatic	(37)	Moderate

Supplementary Table 1: MAP6 partners.

We classified MAP6 partners along the following categories: **Cytoskeleton, Endocytosis, Neuro-receptors & channels, Signal transduction, Nuclear receptors & effectors**. For each partner, the following information is given: Name of the human gene, Aliases, Localizations and roles, Other MAP6 partners found in the same experiment that highlights the partner, Identification method, References of the published work showing the interaction, Confidence of the interaction (Moderate when a single technique was used and with numerous partners found in the study; High when a single technique was used and with only a few partners found in the study; Strong when multiple techniques were used or when multiple independent works exist).

Abbreviations: IF (immunofluorescence assay), NMR (nuclear magnetic resonance), Co-IP (co-immunoprecipitation assay), GST (Glutathione S-transferase).

1. C. Bosc *et al.*, Identification of novel bifunctional calmodulin-binding and microtubule-stabilizing motifs in STOP proteins. *J Biol Chem* **276**, 30904-30913 (2001).
2. C. Delphin *et al.*, MAP6-F is a temperature sensor that directly binds to and protects microtubules from cold-induced depolymerization. *J Biol Chem* **287**, 35127-35138 (2012).
3. J. Lefevre *et al.*, Structural basis for the association of MAP6 protein with microtubules and its regulation by calmodulin. *J Biol Chem* **288**, 24910-24922 (2013).
4. L. Peris *et al.*, A key function for microtubule-associated-protein 6 in activity-dependent stabilisation of actin filaments in dendritic spines. *Nature communications* **9**, 3775 (2018).
5. J. Baratier *et al.*, Phosphorylation of microtubule-associated protein STOP by calmodulin kinase II. *J Biol Chem* **281**, 19561-19569 (2006).
6. F. Letournel, A. Bocquet, F. Dubas, A. Barthelaix, J. Eyer, Stable tubule only polypeptides (STOP) proteins co-aggregate with spheroid neurofilaments in amyotrophic lateral sclerosis. *Journal of neuropathology and experimental neurology* **62**, 1211-1219 (2003).
7. J. C. Deloulme *et al.*, Microtubule-associated protein 6 mediates neuronal connectivity through Semaphorin 3E-dependent signalling for axonal growth. *Nature communications* **6**, 7246 (2015).
8. J. Brocard *et al.*, MAP6 interacts with Tctex1 and Cav 2.2/N-type calcium channels to regulate calcium signalling in neurons. *Eur J Neurosci* **46**, 2754-2767 (2017).
9. L. Sang *et al.*, Mapping the NPHP-JBTS-MKS protein network reveals ciliopathy disease genes and pathways. *Cell* **145**, 513-528 (2011).
10. J. Arama *et al.*, Bmcc1s, a novel brain-isoform of Bmcc1, affects cell morphology by regulating MAP6/STOP functions. *PloS one* **7**, e35488 (2012).
11. S. L. Eastwood *et al.*, Altered expression of synaptic protein mRNAs in STOP (MAP6) mutant mice. *J Psychopharmacol* **21**, 635-644 (2007).
12. D. S. Watkins, J. D. True, A. L. Mosley, A. J. Baucum, Correction: Baucum II, Anthony J. et al. Proteomic Analysis of the Spinophilin Interactome in Rodent Striatum Following Psychostimulant Sensitization. Proteomes 2018, 6, 53. *Proteomes* **7** (2019).
13. S. Gory-Faure *et al.*, Non-microtubular localizations of microtubule-associated protein 6 (MAP6). *PloS one* **9**, e114905 (2014).
14. G. Gorini *et al.*, Dynamin-1 co-associates with native mouse brain BKCa channels: proteomics analysis of synaptic protein complexes. *FEBS Lett* **584**, 845-851 (2010).
15. D. Morderer *et al.*, Endocytic adaptor protein intersectin 1 forms a complex with microtubule stabilizer STOP in neurons. *Gene* **505**, 360-364 (2012).
16. G. E. Craft, M. E. Graham, N. Bache, M. R. Larsen, P. J. Robinson, The in vivo phosphorylation sites in multiple isoforms of amphiphysin I from rat brain nerve terminals. *Molecular & cellular proteomics : MCP* **7**, 1146-1161 (2008).
17. B. M. Schwenk *et al.*, The FTLD risk factor TMEM106B and MAP6 control dendritic trafficking of lysosomes. *EMBO J* **33**, 450-467 (2014).
18. P. N. Vikhreva, E. V. Korobko, I. V. Korobko, Identification of novel proteins, possible interaction partners for guanine nucleotide exchange factor Varp. *Dokl Biochem Biophys* **429**, 323-325 (2009).
19. F. Martins *et al.*, Identification and characterization of the BRI2 interactome in the brain. *Scientific reports* **8**, 3548 (2018).
20. M. A. McFarland, C. E. Ellis, S. P. Markey, R. L. Nussbaum, Proteomics analysis identifies phosphorylation-dependent alpha-synuclein protein interactions. *Molecular & cellular proteomics : MCP* **7**, 2123-2137 (2008).
21. C. S. Muller *et al.*, Quantitative proteomics of the Cav2 channel nano-environments in the mammalian brain. *Proc Natl Acad Sci U S A* **107**, 14950-14957 (2010).
22. H. Kim, S. Jo, H. J. Song, Z. Y. Park, C. S. Park, Myelin basic protein as a binding partner and calmodulin adaptor for the BKCa channel. *Proteomics* **7**, 2591-2602 (2007).
23. K. Lemonidis, M. C. Sanchez-Perez, L. H. Chamberlain, Identification of a novel sequence motif recognised by the ankyrin-repeat domain of zDHHC17/13 S-acyl-transferases. *J Biol Chem* 10.1074/jbc.M115.657668 (2015).
24. E. Tortosa *et al.*, Dynamic Palmitoylation Targets MAP6 to the Axon to Promote Microtubule Stabilization during Neuronal Polarization. *Neuron* **94**, 809-825 e807 (2017).
25. S. J. Won, B. R. Martin, Temporal Profiling Establishes a Dynamic S-Palmitoylation Cycle. *ACS chemical biology* **13**, 1560-1568 (2018).
26. G. P. H. Ho *et al.*, Upregulation of Cellular Palmitoylation Mitigates alpha-Synuclein Accumulation and Neurotoxicity. *Mov Disord* 10.1002/mds.28346 (2020).

27. T. Berggard *et al.*, 140 mouse brain proteins identified by Ca2+-calmodulin affinity chromatography and tandem mass spectrometry. *Journal of proteome research* **5**, 669-687 (2006).
28. S. Yurimoto *et al.*, Identification and characterization of wolframin, the product of the wolfram syndrome gene (WFS1), as a novel calmodulin-binding protein. *Biochemistry* **48**, 3946-3955 (2009).
29. M. E. Capala *et al.*, Mitochondrial Dysfunction in Human Leukemic Stem/Progenitor Cells upon Loss of RAC2. *PloS one* **10**, e0128585 (2015).
30. P. O. Angrand *et al.*, Transgenic mouse proteomics identifies new 14-3-3-associated proteins involved in cytoskeletal rearrangements and cell signaling. *Molecular & cellular proteomics : MCP* **5**, 2211-2227 (2006).
31. B. Wilkinson, J. Li, M. P. Coba, Synaptic GAP and GEF Complexes Cluster Proteins Essential for GTP Signaling. *Scientific reports* **7**, 5272 (2017).
32. M. Del Mar Masdeu *et al.*, Identification of novel Ack1-interacting proteins and Ack1 phosphorylated sites in mouse brain by mass spectrometry. *Oncotarget* **8**, 101146-101157 (2017).
33. K. M. Zoltowska, M. Maesako, O. Berezovska, Interrelationship between Changes in the Amyloid beta 42/40 Ratio and Presenilin 1 Conformation. *Molecular medicine (Cambridge, Mass* **22**, 329-337 (2016).
34. M. Albers *et al.*, Automated yeast two-hybrid screening for nuclear receptor-interacting proteins. *Molecular & cellular proteomics : MCP* **4**, 205-213 (2005).
35. H. Zhang *et al.*, SLX4IP acts with SLX4 and XPF-ERCC1 to promote interstrand crosslink repair. *Nucleic Acids Res* **47**, 10181-10201 (2019).
36. T. Vanderweyde *et al.*, Interaction of tau with the RNA-Binding Protein TIA1 Regulates tau Pathophysiology and Toxicity. *Cell reports* **15**, 1455-1466 (2016).
37. J. Li *et al.*, Spatiotemporal profile of postsynaptic interactomes integrates components of complex brain disorders. *Nat Neurosci* **20**, 1150-1161 (2017).
